# Repositioning Drugs to the Mitochondrial Fusion Protein 2 by Three-Tunnel Deep Neural Network for Alzheimer's Disease

**DOI:** 10.3389/fgene.2021.638330

**Published:** 2021-02-15

**Authors:** Xun Wang, Yue Zhong, Mao Ding

**Affiliations:** ^1^College of Computer Science and Technology, China University of Petroleum, Shandong, China; ^2^Department of Neurology Medicine, The Second Hospital, Cheeloo College of Medicine, Shandong University, Jinan, China

**Keywords:** Alzheimer's disease, drug repositioning, prediction of binding affinity values, three-tunnel deep neural network, molecular docking

## Abstract

Alzheimer's disease (AD) is a common neurodegenerative dementia in the elderly. Although there is no effective drug to treat AD, proteins associated with AD have been discovered in related studies. One of the proteins is mitochondrial fusion protein 2 (Mfn2), and its regulation presumably be related to AD. However, there is no specific drug for Mfn2 regulation. In this study, a three-tunnel deep neural network (3-Tunnel DNN) model is constructed and trained on the extended Davis dataset. In the prediction of drug-target binding affinity values, the accuracy of the model is up to 88.82% and the loss value is 0.172. By ranking the binding affinity values of 1,063 approved drugs and small molecular compounds in the DrugBank database, the top 15 drug molecules are recommended by the 3-Tunnel DNN model. After removing molecular weight <200 and topical drugs, a total of 11 drug molecules are selected for literature mining. The results show that six drugs have effect on AD, which are reported in references. Meanwhile, molecular docking experiments are implemented on the 11 drugs. The results show that all of the 11 drug molecules could dock with Mfn2 successfully, and 5 of them have great binding effect.

## 1. Introduction

Alzheimer's disease (AD) is a destructive nervous system disease, which is characterized by a progressive dementia. The incidence of AD accounts for 50–70% of the total number of senile dementias. It mostly occurs in middle or late life, and the psychological skills, cognitive function, and physiological function of the patients have gradually lost (McKhann et al., 1984; Navarro et al., 2020). With the increasing aging of the population, AD has become an important world problem to be solved. However, the pathogenesis of AD is still unclear. The cascade hypothesis of amyloid β protein (Aβ) is the most concerned. The hypothesis holds that the formation of senile plaques by a large amount of Aβ in the brain is related to cognitive dysfunction and pathological changes of AD (Lin Zhang et al., 2020). Abnormal deposition of Aβ is considered to be the vital pathogenesis of AD. And Aβ in cerebrospinal fluid has been included as a diagnostic marker of AD (Jia and Wei, 2018; Cui et al., 2020). A variety of AD-targeted drugs are difficult to be used in clinical practice because of poor efficacy or side effects in phase III clinical trials. More scholars focus on controlling the progression of mild cognitive impairment. Consequently, the regulation mechanism of Aβ production and clearance has become an important research direction (Cui et al., 2020).

Mitochondria is the main site of cellular aerobic respiration. And mitochondrial dysfunction has effect on the production and toxicity of Aβ. Mitochondrial dynamics and mitochondrial dysfunction caused by abnormal mitochondrial autophagy play an important role in the pathogenesis of AD. The mitochondrial fusion protein 2 (Mfn2) is a dynamic protein expressed in the outer membrane of mitochondria. Mfn2 not only participates in mitochondrial fusion but also affects cell metabolism by regulating cell apoptosis, mitochondrial autophagy, and other biological processes. At present, Mfn2 has been proved to be closely related to the occurrence of many kinds of common diseases. Although some specific mechanisms are still unclear, Mfn2 is expected to become a new therapeutic target for some diseases (Li et al., 2020). In addition, Mfn2 involved in the regulation of protein homeostasis and pathogenesis of AD has become a research hotspot.

The approved drugs are designed and developed based on the concept of single target. Therefore, no drug has been specifically developed for Mfn2 regulation till now. It takes 10–15 years to implement the *de novo* drug design. In order to reduce the cost of drug development and the risk in the process of drug research, it has become an important strategy to repurpose the approved drugs and explore their new functions. And deep learning methods provide powerful technical supports in computing the drug-target interactions (DTIs). The prediction of DTIs is the focus of drug design and the key step of drug repositioning. However, it is obviously not accurate to divide the drug–target pairs (DT pairs) into effective and ineffective in the classification method. Therefore, more attention has been paid to the regression method, which directly predicts the binding affinity values of drug–target (DT) pairs with dissociation constant (K_*d*_).

The DeepDTA model (Ozturk et al., 2018) considers the sequence information of drug molecules and proteins in the prediction of binding affinity values. Convolutional neural network is used in the research. It is considered to be the state-of-the-art model of predicting DTA (Huang et al., 2020). However, the model fluctuates greatly when training for many times. The GraphDTA model (Nguyen et al., 2020) uses graph convolution neural network to represent the features of drug molecules. Although its loss value is tiny, the calculation cost is too high. Recurrent neural networks (RNNs) such as gated recurrent units (GRU) (Cho et al., 2014) and long short-term memory units (LSTM) (Hochreiter and Schmidhuber, 1997) are widely used to capture temporal dependence in sequence-based data such as time series and text (Chuang et al., 2020). Extending on the use of a single RNN, the ensemble of RNNs with CNNs is a common hybrid architecture in recent applications that seeks to combine the ability of RNN in analyzing sequential data and CNN on extracting local features (Cao et al., 2020). Nonetheless, in the representation of drug molecules, the results are not better than that of CNN. The latest DeepGS model (Lin et al., 2020) inputs the sequence information and two-dimensional structure information of drug molecules as well as the protein sequence information into the model for prediction. It also has the problem of higher calculation cost. Moreover, information redundancy is inevitable as drug molecules are encoded twice by different encoding strategies. In addition, DeepPurpose (Huang et al., 2020) provides a toolkit that integrates a variety of encoding methods of drug molecules and protein amino acid sequences. Two kinds of encoding methods are selected to input the model to predict the binding affinity values of DT pairs. The toolkit provides great convenience for future research.

In this study, we implement an approach that considers the binding affinity information and negative samples of DT pairs to reposition regulatory drugs Mfn2 as candidate medications of AD. First, a three–tunnel deep neural network (3-Tunnel-DNN) model is constructed and trained on the expanded Davis dataset using drug–protein binding affinity information. The three tunnels are protein sequences, drug molecules of positive samples, and negative samples. The accuracy of the 3-Tunnel-DNN model is 0.8882 and the loss value is 0.172 in the test set. Finally, the well-trained model is used to reposition 1,063 drugs/compounds from the DrugBank database to Mfn2 regulatory. A total of 15 drugs are recommended for Mfn2 regulation by ranking the binding affinity values of drugs/compounds from the database with Mfn2. After removing three molecules with molecular weight <200 and a topical drug, a total of 11 drug molecules are selected for literature mining and molecular docking experiments.

## 2. Materials and Methods

### 2.1. The Extended Davis Dataset

Davis dataset contains the selective analysis of kinase protein family and related inhibitors and their respective K_*d*_ values, and it includes 30,056 binding affinity values of 442 proteins and 68 compounds (Ozturk et al., 2018; Davis et al., 2020). Negative samples are expected to be considered in our model. Davis dataset is widely used as training set in the field of drug-target binding affinity prediction, such as DeepDTA (Ozturk et al., 2018), DeepGS (Lin et al., 2020), GraphDTA (Nguyen et al., 2020), etc. Therefore, binding affinity values of DT pairs in Davis dataset are applied as training set in the 3-Tunnel DNN model as well. Besides, information of negative samples is added into Davis dataset to extend the dataset.

In the original Davis dataset, binding affinity data of DT pairs are measured by K_*d*_ values. It ranges from 0 to 10,000. The extended Davis dataset consists of four files, which are SMILES sequences file of compounds, FASTA sequences file of proteins, binding affinity values file of DT pairs, and SMILES sequences file of negative samples. The original Davis dataset consists of the first three files. The first and second files contain the sequence information needed in the model training process. The third file, in particular, is a 68 × 442 dimensional digital matrix [i.e., M_(68 × 442)_], in which each number [m_(*i, j*)_] represents the K_*d*_ value of the i-th compound and the j-th protein. The fourth file is a matrix [i.e., NM_(68 × 442)_] composed of SMILES sequences of negative samples. Each element [nm_(*i, j*)_] represents the SMILES sequence of the negative sample of the i-th compound and the j-th protein. In the research, the K_*d*_ value of 50 is taken as the boundary between positive and negative samples. It means that for each protein, the compounds with binding affinity value ≤ 50 are positive samples, and the compounds with >50 are negative samples. The extended Davis dataset is given in Supplementary Tables 1–4.

In fact, we compare the recommendation of Zeng et al. that the boundary of positive and negative samples is set as 10 (Zeng et al., 2019) with our boundary of 50 as well. The results show that the K_*d*_ value of 50 as the boundary performs better. The results are shown in Table 1.

**Table 1:** Comparison list of consistency index (CI) and mean square error (MSE) of different boundaries on test set.

**Model**	**Mse**	**CI**
CNN_CNN (boundary is set as 10)	0.878	0.261
*CNN_CNN (boundary is set as 50)*	*0.881*	*0.245*
3-Tunnel DNN (boundary is set as 10)	0.882	0.252
***3-Tunnel DNN (boundary is set as 50)***	***0.888***	***0.172***

*Italic text indicates a better boundary between positive and negative samples. Bold text represents the best performance*.

In the training process, the K_*d*_ values converted into log space (pK_*d*_) are used as the actual binding affinity values for easier calculation of regression. The explanation is similar to Equation (1) (Huang et al., 2020).

(1) $$\begin{array}{l} pK_{d}=-log_{10}\left(K_{d}\times 10^{-9}+10^{-10}\right) \end{array}$$

**Figure 1:**
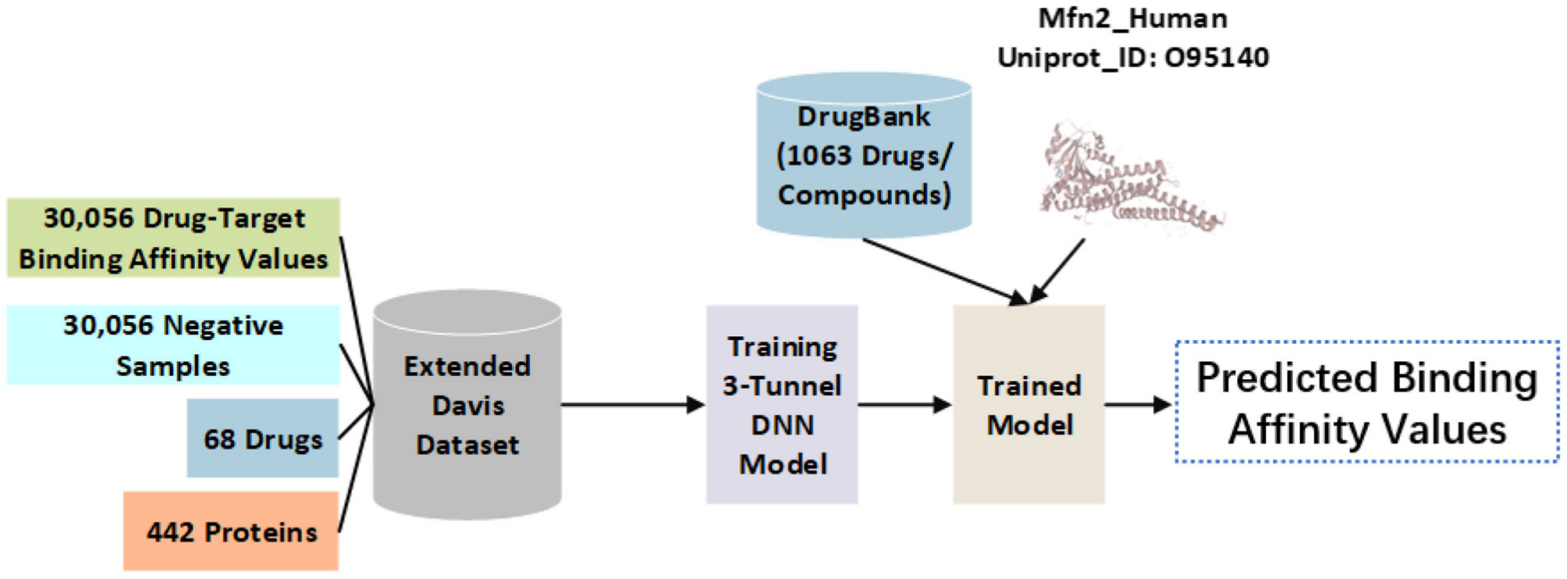
The use of data in the model. Binding affinity values of DT pairs in the extended Davis dataset are used for training set. The 1,063 drugs/compounds from the DrugBank database and Mfn2_Human from the UniProt database are used for external test set. Binding affinity values among drugs and Mfn2_Human are predicted using the well-trained model to recommend repositioning regulatory drugs to Mfn2_Human.

The DrugBank database contains common compounds (amino acids, polypeptides, choline, etc.), approved drugs (azithromycin, etc.), and approved small molecular compounds (5-fluorouridine, etc.). The first 1,063 drugs/compounds in the DrugBank database are used here as potential candidates for repositioning regulatory Mfn2. Particularly, SMILES sequences of drugs are used for calculation. Mfn2 sequence of human protein (Mfn2_Human) is used as the target protein for repositioning. Mfn2_Human in form of FASTA sequence from the UniProt database is used for binding affinity calculations. The usage of data is shown in Figure 1.

### 2.2. Feature Extraction of Drug Molecules and Proteins

Extended-connectivity fingerprints (ECFPs) are a novel class of topological fingerprints for molecular characterization, which is a 1,024-length bits vector (Rogers and Hahn, 2010). In the study, *n=2* (i.e., ECFP_2) is chosen as the circular radius that encodes the substructure of drug molecules. RDKit (Bento et al., 2020) is used to generate fingerprints of molecules. A multi-layer perceptron (MLP) (Chuang et al., 2020) is then applied on the binary fingerprint vector (Huang et al., 2020). In the 3-Tunnel DNN model, MLP is constructed as a four-layer neural network that the number of neurons is 1,024, 256, 64, and 256, respectively, to extract feature representations of drug molecules.

For proteins, there are 25 unique characters in protein FASTA sequence in Davis dataset (Ozturk et al., 2018). In our model, the symbol “?” is filled in the beginning of each sequence (Huang et al., 2020). Therefore, there are 26 unique characters in FASTA sequences. Each character is mapper into a unique integer, and the FASTA sequences are transformed into one-dimensional vectors. After that, the vector is extended into square data structure, in form of binary matrix with one-hot encoding strategy. The maximum length of FASTA sequences is set as 1,000 (Ozturk et al., 2018), so the matrix size of FASTA sequences is “1,000 × 26.” In particular, if the length of FASTA sequence is <1,000, the matrix is filled with 0. The matrix is input into the convolutional neural network (CNN), which consists of three layers of one-dimensional convolutional network and a global maximum pooling layer. The convolutional kernel is 32 × 1, 32 × 2, and 32 × 3, respectively (Ozturk et al., 2018; Huang et al., 2020). The activation function is Rectified Linear Unit (ReLU) (Nair and Hinton, 2010). And then, the representation vector of the features is generated.

**Figure 2:**
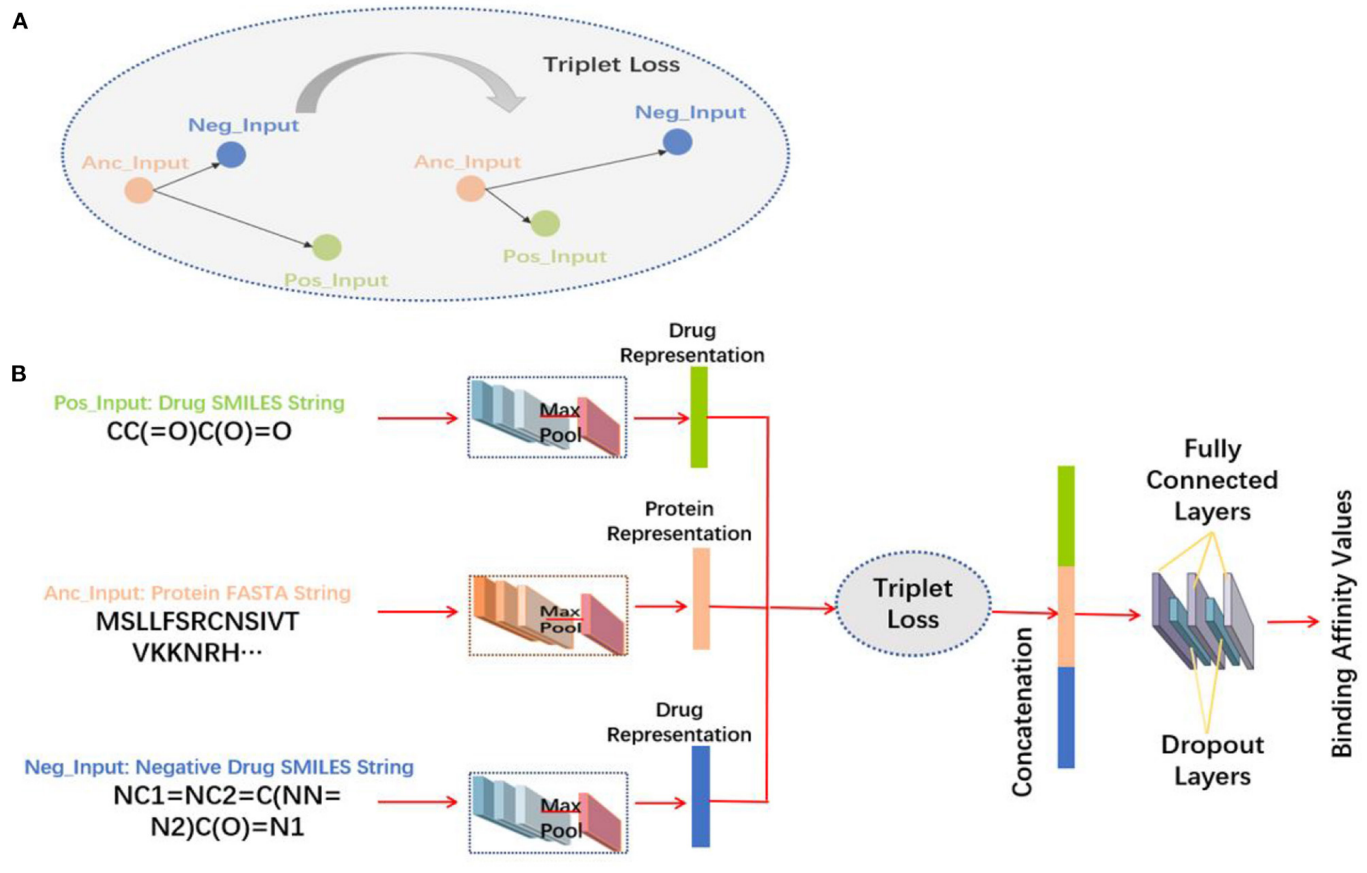
Structure diagram of the 3-Tunnel DNN model. **(A)** Diagram of triplet loss and **(B)** flowchart of the 3-Tunnel DNN model.

The feature extraction methods are shown in Figure 2B.

### 2.3. 3-Tunnel DNN Model

Information of negative samples is expected to be considered in the process of model training. Therefore, on the basis of two tunnels of drug molecules and proteins amino acid sequences, the third tunnel is added to process the data of negative samples to accurately reposition the drugs. The amino acid sequence of the protein is set as the anchor input (Anc_Input), the positive samples of the extended Davis dataset are taken as positive input (Pos_Input), and negative samples are set as negative input (Neg_Input). In the process of learning feature representations, triplet loss (Davis et al., 2020) is used to minimize the distance between Anc_Input and Pos_Input, and maximize the distance between Anc_Input and Neg_Input. The triplet loss is explained in Equation (2).

(2) $$\begin{array}{l} L=max(\|f(X_{i}^{Anc\_input})-f(X_{i}^{Pos\_input}){\|}_{2}^{2}-\|f(X_{i}^{Anc\_input})\\ \;-f(X_{i}^{Neg\_input}){\|}_{2}^{2}+M,0) \end{array}$$

where $f\left(X_{i}^{Anc\text{\_}input}\right)$ represents the feature representation of the *i*th protein amino acid sequence, $f\left(X_{i}^{Pos\text{\_}input}\right)$ represents the feature representation of the *i*th positive sample, while $f\left(X_{i}^{Neg\text{\_}Input}\right)$ represents the feature representation of the *i*th negative sample. In addition, $∥f\left(X_{i}^{Anc\text{\_}input}\right)-f\left(X_{i}^{Pos\text{\_}input}\right){∥}_{2}^{2}$ means the square of the Euclidean distance between the vectors of the *i*th Anc_Input and Pos_Input. Similarly, $∥f\left(X_{i}^{Anc\text{\_}input}\right)-f\left(X_{i}^{Neg\text{\_}input}\right){∥}_{2}^{2}$ means the square of the Euclidean distance between the vectors of the *i*th Anc_Input and Neg_Input. And M is a hyperparameter, which is set to 1 in the manuscript.

The three tunnels are used to process the FASTA sequences of proteins, the SMILES sequences of positive samples, and negative samples of drug molecules, respectively. The triplet loss (Schroff et al., 2015) is used here to obtain more accurate feature representations by maximizing the distance between proteins and negative samples and minimizing the distance between proteins and negative samples (Figure 2A). And then, three feature representations are concatenated together and input into the fully connected layers to make nonlinear changes to these extracted feature representations. In particular, the first two fully connected layers are followed by a dropout layer, respectively, which randomly “delete” hidden neurons to prevent over fitting, and finally map to the output space. The output of the model is the predicted binding affinity values of DT pairs. The 3-Tunnel DNN model is based on the MLP_CNN model (MLP for drugs encoding, CNN for proteins encoding) in DeepPurpose toolkit (Lin et al., 2020), and its topologic structure is shown in Figure 2B.

The 30,056 DT pairs from the extended Davis dataset are taken as training set, which are divided into three subsets in the ratio of 7:1:2 (Huang et al., 2020). It means that 70% of the data are used for training, 10% for validation, and 20% for testing. We use 256 small batch data to update the weights of neural networks. The number of epochs of the 3-Tunnel DNN model is 100, as well Adam optimization algorithm (with learning rate of 10^−4^) is applied to optimize the model.

### 2.4. Drug Reposition of Mfn2 by Well-Trained 3-Tunnel DNN Model

After the well-trained 3-Tunnel DNN model is saved, 1,063 SMILES sequences of drugs/compounds from the DrugBank database and Mfn2_human protein sequence in the form of FASTA sequence from the UniProt database are input into the well-trained model. These predicted values are ranked to get the drug recommendation list. After removing the molecules with molecular weight <200 and topical drugs, a total of 11 drug molecules are recommended to regulate Mfn2. Literature mining and molecular docking experiments are implemented to verify the effectiveness of these molecules.

### 2.5. Molecular Docking

A total of 11 structures of recommended drug molecules and Mfn2 are analyzed by molecular docking experiments. The X-ray crystal structure of Mfn2 (PDB code: 6JFK, Resolution: 2.00 Å) is downloaded from RCSB Protein Data Bank (http://www.rcsb.org) in PDB format, and the first conformation is chosen as the receptor structure. The three-dimensional structures of recommended molecules are downloaded from the DrugBank database (https://www.drugbank.ca) in PDB format as well. UCSF Chimera software (Pettersen et al., 2004) is used to prepare receptor protein binding sites, establish the three-dimensional structure of molecules, and minimize the energy.

Before the formal docking experiments, it is necessary to prepare the documents of receptor protein, binding sites, protein surface, and drug molecules. For the receptor protein, all structures of ligands and hydrogens are deleted first. Dock Prep module is used to supplement the parameters of the receptor protein. Hydrogens, AMBER ff14SB force field, and AM1-BCC charges (Jakalian et al., 2000, 2010) of receptor and ligand are added, respectively. After that, the results are saved to a file in mol2 format. Then all hydrogens are deleted again and saved in PDB format. For the binding sites in the receptor, the same operation is implemented and the result is saved in the format of mol2. The DMS tool in UCSF Chimera (Pettersen et al., 2004) is used to generate the surface of the receptor using a probe atom with a 1.4 Å radius, which is saved as the file in the format of dms. Similarly, for drug molecules, hydrogenation, and charging operations are performed, and the results are saved as a file in the format of mol2 as well.

For each binding site, the Sphgen module is used to generate a spherical collection around the active site. The grid file is generated by the Grid module, which is used for grid-based energy assessment. Then, the semi-flexible docking is implemented with the program of Dock6.8 (Lang et al., 2009; Mukherjee et al., 2010), and 1,000 different orientations are generated. In particular, some drug molecules (e.g., adinazolam, pyrimethamine, and carbamazepine) are implemented rigid molecular docking to evaluate whether the receptor can accommodate the conformation. After that, the van der Waals force and electrostatic interaction between the ligand and the binding site are obtained, and the grid scores are also calculated. Finally, the best conformation is obtained by using cluster analysis (RMSD threshold is 2.0 Å) in semi-flexible docking, and in rigid docking, only one conformation is obtained.

## 3. Results

### 3.1. Results of Model Training

In the training process, consistency index (CI) (Pahikkala et al., 2014) is used to evaluate the training performance, and mean square error (MSE) (Kansal et al., 2019) is used as the loss function to measure the error of each epoch.

**Figure 3:**
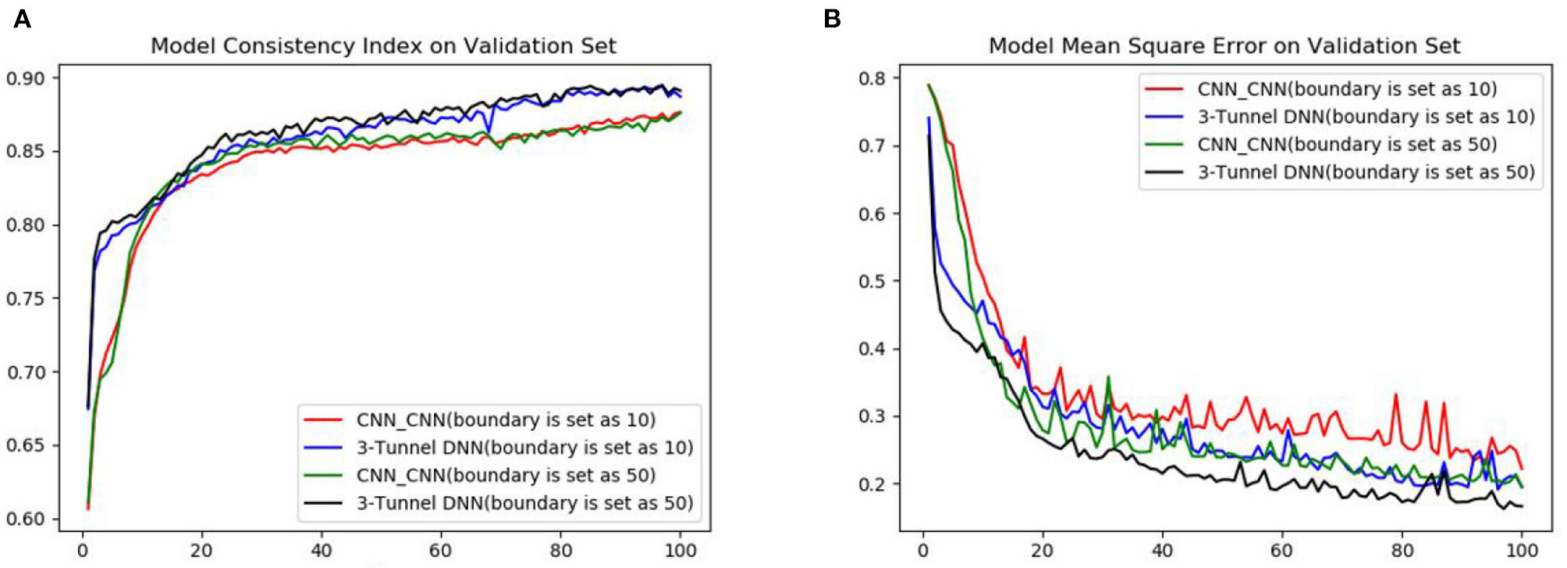
Comparison diagram of different boundaries in CNN_CNN model and MLP_CNN model. **(A)** Diagram of the accuracy on validation set and **(B)** diagram of the loss on validation set.

We compare the performance of boundary with the K_d value of 10 (Zeng et al., 2019) and 50 as positive and negative samples. The performance of CNN_CNN model (CNN for drugs encoding, CNN for proteins encoding) and the 3-Tunnel DNN model at different boundaries are compared primarily. The results are shown in Table 1 and Figure 3. The results show that the K_d value of 50 as the boundary of positive and negative samples makes the model perform better. And our 3-Tunnel DNN model performs better than CNN_CNN model.

The 3-Tunnel DNN model is compared with the performance with the state-of-the-art model at present, such as DeepDTA (Ozturk et al., 2018), GraphDTA (Nguyen et al., 2020), and the latest DeepGS (Lin et al., 2020) model. At the same time, the original CNN_CNN model (Huang et al., 2020) and other models obtained in the DeepPurpose toolkit using our extended Davis dataset, such as CNN_CNN, CNN+LSTM_CNN (CNN+LSTM for drugs encoding, CNN for proteins encoding), and CNN+GRU_CNN (CNN+GRU for drugs encoding, CNN for proteins encoding) model, are also compared with the 3-Tunnel DNN model. The performances of these models are shown in Table 2.

**Table 2:** Comparison list of consistency index (CI) and mean square error (MSE) of different models on test set.

**Model**	**Mse**	**CI**
CNN_CNN (boundary is set as 10)	0.878	0.261
DeepDTA (Davis dataset) (Ozturk et al., 2018)	0.878	0.261
GraphDTA (Davis dataset) (Nguyen et al., 2020)	0.881	0.245
DeepGS (Davis dataset) (Lin et al., 2020)	0.882	0.252
CNN_CNN (Davis dataset) (Huang et al., 2020)	0.879	0.254
CNN_CNN (our extended Davis dataset)	0.87	0.209
CNN+LSTM_CNN (our extended Davis dataset)	0.86	0.234
CNN+GRU_CNN (our extended Davis dataset)	0.834	0.263
**3-Tunnel DNN (our extended Davis dataset)**	**0.888**	**0.172**

*Bold text represents the best performance*.

According to results of the 3-Tunnel DNN model, the value of CI on test set is 0.888 and that of MSE is 0.172, which performs best among these models. The CI value of the 3-Tunnel DNN model is improved by 0.6% compared with DeepGS model (Lin et al., 2020), that is, with best accuracy. And the MSE value is improved by 7.3% compared with GraphDTA model (Nguyen et al., 2020), that is, with minimum loss. In addition, we also find that the MSE value of models using the extended Davis dataset as the training set is significantly smaller than models trained on the original Davis dataset, except for CNN+GRU_CNN model.

### 3.2. Results of Recommended Repositioning Regulatory Drugs to Mfn2

For repositioning regulatory drugs to Mfn2, the well-trained 3-Tunnel DNN model is used to calculate the binding affinity value of each pair of potential drug-Mfn2 pairs. The SMILES of potential drugs are the 1,063 approved drugs in the DrugBank database, and Mfn2 is the amino acid sequence of human in the form of FASTA sequence from the UniProt database. According to the ranking of predicted values, the 11 drugs recommended by 3-Tunnel DNN model are shown in Table 3 after removing drugs with molecular weight <200 (niacin, ethionamide, and acetohydroxamic acid) and an anesthetic (dyclonine). In particular, although dyclonine is reported to be effective for targets of AD in the reference (Zhang et al., 2019), it is still removed from the results because of topical drug.

**Table 3:** Recommended drugs by the three-tunnel deep neural network (3-Tunnel DNN) model.

**Drug name**	**Drug ID**	**Disease treated with the drug**	**References & Descriptions**
Imatinib	DB00619	Antineoplastic	It is confirmed that imatinib-mediated control of neprilysin could indeed be accounted for its effect on activation induced cell death (Bauer et al., 2011)
Lamotrigine	DB00555	Antiepileptic	The study shows that lamotrigine is an effective and safe monotherapy in patients with cognitive disorders and AD (Tsolaki et al., 2000)
Sulfametopyrazine	DB00664	The treatment of respiratory, urinary tract infections, and malaria	
Adinazolam	DB00546	Anxiolytic, anticonvulsant, sedative, and antidepressant	
Pyrimethamine	DB00205	Antimalarial or the treatment of toxoplasmosis	
Bosentan	DB00559	The treatment of pulmonary hypertension	The study shows that bosentan can play a pathophysiological role in the endogenous endothelin system of AD (Elesber et al., 2006)
Voriconazole	DB00582	Antifungal	
Fluphenazine	DB00623	The treatment of psychoses	Fluphenazine and other depot neuroleptics are used in AD patients to treat behavioral disorders (Gottlieb et al., 1988)
Pemetrexed	DB00642	Antineoplastic	
Nabumetone	DB00461	Anti-inflammatory	It provides a method for treating and preventing dementia such as AD, which comprises administering an effective, nontoxic amount of nabumetoneor 6MNA (Clark, 1997)
Carbamazepine	DB00564	Anticonvulsant and analgesic	The result shows that carbamazepine may be useful to treat agitated AD patients (Gleason and Schneider, 1990)

We search the keywords “Alzheimer; Drug name” and find that 6 drugs have supported references. Names, DrugBank ID, original functions, and description of supported references of these drugs are shown in Table 3.

It is reported that neuroleptic medication appears to have modest efficacy in controlling behavioral symptoms in dementia patients (Lemke, 1995). And the three drugs (adinazolam, fluphenazine, and carbamazepine) are related to mental illness. In addition, anti-tumor drugs, anti-epilepsy drugs, anti-infection drugs, and drugs for the treatment of hypertension are included in the recommended list.

### 3.3. Results of Molecular Docking

Dock6.8 program (Lang et al., 2009; Mukherjee et al., 2010) is used to predict the binding patterns of 11 drug molecules in Mfn2. The value of Grid_Score is used to evaluate the molecular docking results, which represents the sum of van der Waals force and electrostatic interaction. The negative value of Grid_Score indicates that the drug molecule is bound to the target, while the positive value indicates no binding. And the smaller the Grid_Score, the stronger binding of drug molecules to Mfn2. Generally, the value of Grid_Score >−40 kcal/mol indicates poor binding, the value between −40 and −50 kcal/mol indicates medium binding, and the value < −50 kcal/mol indicates great binding (Liu et al., 2018). The Grid_Score values of each drug molecule binding to Mfn2 are shown in Table 4.

**Table 4:** Results of molecular docking.

**Drug name**	**Drug ID**	**Grid score**	**Is supported by references**
**Imatinib**	**DB00619**	**−51.678650**	**Yes**
Lamotrigine	DB00555	−28.860310	Yes
Sulfametopyrazine	DB00664	−43.703491	No
*Adinazolam*	*DB00546*	*−29.337458*	*No*
*Pyrimethamin*	*DB00205*	*−31.183176*	*No*
**Bosentan**	**DB00559**	**−55.177814**	**Yes**
Voriconazole	DB00582	−39.047768	No
Fluphenazine	DB00623	−49.189960	Yes
**Pemetrexed**	**DB00642**	**−54.729557**	**No**
Nabumetone	DB00461	−38.957726	Yes
*Carbamazepine*	*DB00564*	*−34.177979*	*Yes*

*Bold text shows the drugs with Grid_Score < −50 kcal/mol, and italic text represents rigid molecular docking*.

According to the results of molecular docking, the binding effect of bosentan and Mfn2 is the best, and it is supported by the reference (Elesber et al., 2006) as well. In addition, the Grid_Score of imatinib and pemetrexed are < −50 kcal/mol, and they also have strong binding with Mfn2. Pemetrexed is not supported by any reference, but its binding capacity to Mfn2 is slightly lower than bosentan. And sulfametopyrazine and fluphenazine have medium binding with Mfn2. However, lamotrigine, voriconazole, and nabumetone have poor binding with Mfn2 in the experiments of semi-flexible docking. Lamotrigine, in particular, has the lowest score among these drug molecules, although it is supported by reference (Tsolaki et al., 2000). In addition, the Grid_Scores of adinazolam, pyrimethamine, and carbamazepine are not satisfactory. We speculate that it is caused by the rigid molecular docking. Since atomic bonds cannot rotate, there is only one conformation in the rigid docking.

## 4. Discussion

In this study, we construct a 3-Tunnel DNN model based on the original drug-target binding affinity prediction model to consider the influence of negative samples. The binding affinity values of DT pairs are trained on the extended Davis dataset (i.e., the positive and negative samples are divided by the K_d value of 50 on the original Davis dataset). Then, the binding affinity values of 1,063 drug molecules with Mfn2 protein are predicted using a well-trained deep learning model. Literature mining and molecular docking experiments are implemented on the recommended 11 molecules. The values of accuracy and loss of the model are obviously better than the existing models, especially the loss value is 0.172. Six of the 11 molecules have been reported by other researchers. The results of molecular docking show that all of the 11 drug molecules can dock with Mfn2 successfully. And five drug molecules have medium or strong binding force. In particular, bosentan has the best performance of molecular docking, which is also supported by the reference (Elesber et al., 2006). In addition, pemetrexed and imatinib are prospect drugs as well. Specially, pemetrexed has not been used in the treatment of AD, and its molecular docking result is just tiny lower than bosentan. In the following work, we will evaluate the pharmacology and toxicology of pemetrexed, and *in vitro* experiments could be prepared to verify its effectiveness.

Although we find that positive and negative samples in Davis dataset with the K_d value of 50 as the boundary is better than 10, its specific value is still worthy to study. In addition, other datasets (such as KIBA and BindingDB) are expected to be extended and implemented in the model in our future work. And, it is a promising research to add gene information (Chen et al., 2013; Zhang et al., 2013; Shi et al., 2014; Tan et al., 2014) into the drug–target relationships and explore the relationships between genes (Li et al., 2015, 2016; Shi et al., 2015) and drugs. Furthermore, the training speed and accuracy of the feature representations of drug molecules extracted by molecular fingerprint is obviously better than that of SMILES sequences. For further research, a better feature extraction method for protein characteristics is expected to be obtained. And spiking neural P systems (Song et al., 2013, 2015a,b; Song and Pan, 2015) is also considered to be implemented in the future.

## Data Availability Statement

The original contributions presented in the study are included in the article/Supplementary Material, further inquiries can be directed to the corresponding author/s.

## Author Contributions

XW conceived and designed the experiments. YZ performed the experiments and wrote the code. MD analyzed the data. XW and YZ drafted the work and revised it. All authors contributed to the article and approved the submitted version.

## Conflict of Interest

The authors declare that the research was conducted in the absence of any commercial or financial relationships that could be construed as a potential conflict of interest.

## Supplementary Material

Supplementary Tables 1–4 The extended Davis dataset.
